# Novel Coronavirus, Old Partisanship: COVID-19 Attitudes and Behaviours in the United States and Canada

**DOI:** 10.1017/S0008423920000463

**Published:** 2020-05-12

**Authors:** Mark Pickup, Dominik Stecula, Clifton van der Linden

**Affiliations:** 1 Department of Political Science, Simon Fraser University, 8888 University Drive AQ 6039, Burnaby, British Columbia, V5A 1S6; 2 Department of Political Science, Colorado State University, 1782 Campus Delivery, Fort Collins, Colorado 80523, USA; 3 Department of Political Science, McMaster University, 1280 Main Street West, Hamilton, Ontario L8S 4M4

## Abstract

The novel coronavirus reached the United States and Canada almost at the same time. The first reported American case was January 20, 2020, and in Canada it was January 15, 2020 (Canada, 2020; Holshue et al., 2020). Yet, the response to this crisis has been different in the two countries. In the US, President Donald Trump, prominent Republicans, and conservative media initially dismissed the dangers of COVID-19 (Stecula, 2020). The pandemic became politicized from the early days, and even though Trump and Republicans have walked back many of their initial claims, there continue to be media reports of partisan differences in public opinion shaped by that early response. At the same time, the response in Canada has been mostly characterized by across-the-board partisan consensus among political elites (Merkley et al., 2020).

The novel coronavirus reached the United States and Canada almost at the same time. The first reported American case was January 20, 2020, and in Canada it was January 15, 2020 (Canada, 2020; Holshue et al., 2020). Yet, the response to this crisis has been different in the two countries. In the US, President Donald Trump, prominent Republicans, and conservative media initially dismissed the dangers of COVID-19 (Stecula, 2020). The pandemic became politicized from the early days, and even though Trump and Republicans have walked back many of their initial claims, there continue to be media reports of partisan differences in public opinion shaped by that early response. At the same time, the response in Canada has been mostly characterized by across-the-board partisan consensus among political elites (Merkley et al., 2020).

Despite vastly different initial responses by political elites in the US and Canada, we know relatively little about how the publics in both countries compare. In this article, we examine nationally representative survey data from the US and Canada, fielded in late March and early April, to examine American and Canadian attitudes in relation to topics including concern about COVID-19, evaluations of the federal government's response to the pandemic, and confidence in the federal government's ability to handle it, as well as self-declared changes in behaviours recommended by public health experts to combat COVID-19 (such as avoiding crowds and social distancing). We examine these attitudes and behaviours through the prism of partisanship, and whether partisan divisions pose risks to public health in the US and Canada by reducing responses to COVID-19 needed to fight the pandemic.

## Partisanship and Polarization of COVID-19 Opinion

Public opinion is shaped, to a large extent, by political elites (Lenz, 2012; Zaller, 1992). At a time of high political polarization, especially in the United States, partisan identities are very salient (Mason, 2018), and, as a result, when trusted political leaders engage on a prominent issue, the public tends to follow. This dynamic applies to both “political” phenomena and scientific issues, such as climate change (Merkley and Stecula, 2020) and potentially COVID-19.

At the time of writing, the novel coronavirus global pandemic is extremely salient. Based on public opinion polling, an unprecedented 89 per cent of Americans followed the COVID-19 news closely in March (Mitchell and Oliphant, 2020). Polls in Canada show a similar trend, with three-quarters of Canadians closely following news or information about the outbreak (Abacus, 2020). With the vast majority of Americans and Canadians paying close attention to COVID-19 news, the messages of political elites are of special importance. Given differences in the degree to which political elites have politicized the issue in the US and Canada, we expect to find higher opinion polarization in the United States. This is important to the extent that it might affect behaviours needed to fight the pandemic.

## Data and Methods

The data employed in this study are sourced from two surveys. The American survey was fielded through Lucid on March 31, 2020 (*n* = 1,009). We weight on whether the respondent is Hispanic or not, White or not, and educational attainment (see Supplementary Materials, available online, for sampling details). Canadian data come from Vox Pop Labs COVID-19 Monitor initiative, collected between March 20 and April 7, 2020 (*n* = 9,889). Weights are based on age group, sex, highest level of educational attainment, vote recall in the 2019 Canadian federal election, and region.

Our dependent variables are COVID-19 attitudes and behaviours (see the Supplementary Materials for question wording). Respondents were asked how concerned they are about the pandemic^1^ (*COVID-19 Concern*); whether they believed that the actions being taken by the federal government in response to coronavirus were an overreaction, an underreaction, or appropriate (*Government Reaction*); how confident they are in the federal government's ability to safeguard the health and well-being of the American/Canadian people during the pandemic (*Government Confidence*); and what changes they have made to their normal routine in response to coronavirus^2^ (*COVID-19 Behaviours*).

Our independent variable is partisanship. In the American sample, this is based on a question asking if the respondent identified as a Republican or Democrat. In the Canadian sample this is based on reported vote in the 2019 federal election.

*COVID-19 Concern*, *Government Reaction*, and *Government Confidence* have categorical response options. We tested for partisan differences using multinomial logit, controlling for demographic variables, and then calculating the average probability of being in each response category holding demographic variables at their observed values for each respondent.^3^ We then tested the statistical significance of differences in probabilities across partisanship. For *COVID-19 Behaviours*, we calculated the proportion of all possible behaviours each respondent indicated he or she had engaged in and used a linear regression controlling for demographic variables. The demographic variables are age, gender, income, language (Canada only), education, region, and ethnicity (US only).^4^

## Results

**Figure 1.**
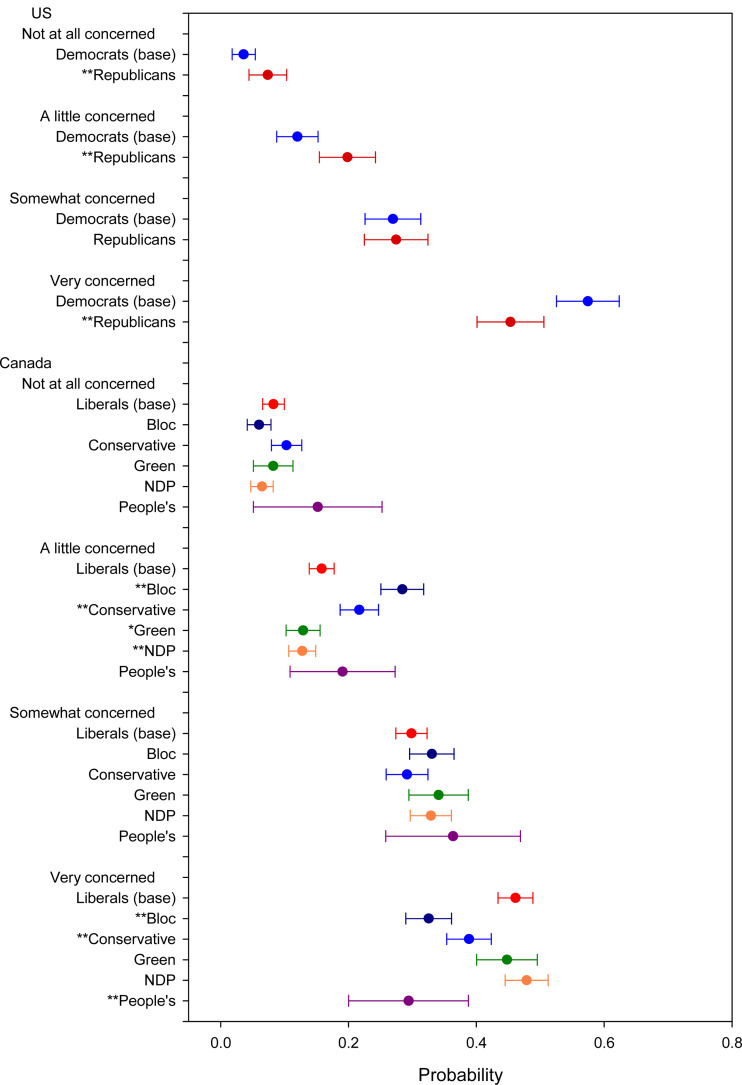
COVID concern. Note: *significant at 0.1 level; **significant at 0.05 level.

Starting with *COVID-19 Concern* (Figure 1), we see that Democrats are more likely to say they are very concerned (57%) compared to Republicans (43%). The difference is statistically significant at the 0.05 level.^5^ Consequently, Democrats are comparatively less likely to say they are only a little concerned or not at all concerned. For the Canadian case, we compare Liberal supporters (the governing party) to supporters of each of the other parties. We do not see much of a difference between Liberal and Green or NDP supporters. However, Conservatives are somewhat less likely to say they are very concerned (39%) compared to Liberals (46%), and Bloc supporters are even less likely to be very concerned (33%) and more likely to be a little concerned. These differences are statistically significant. Peoples Party (PPC) supporters are the most different from Liberals, only 29 per cent of whom indicated they were very concerned. This 17-percentage-point difference is similar to that between Republicans and Democrats.

**Figure 2.**
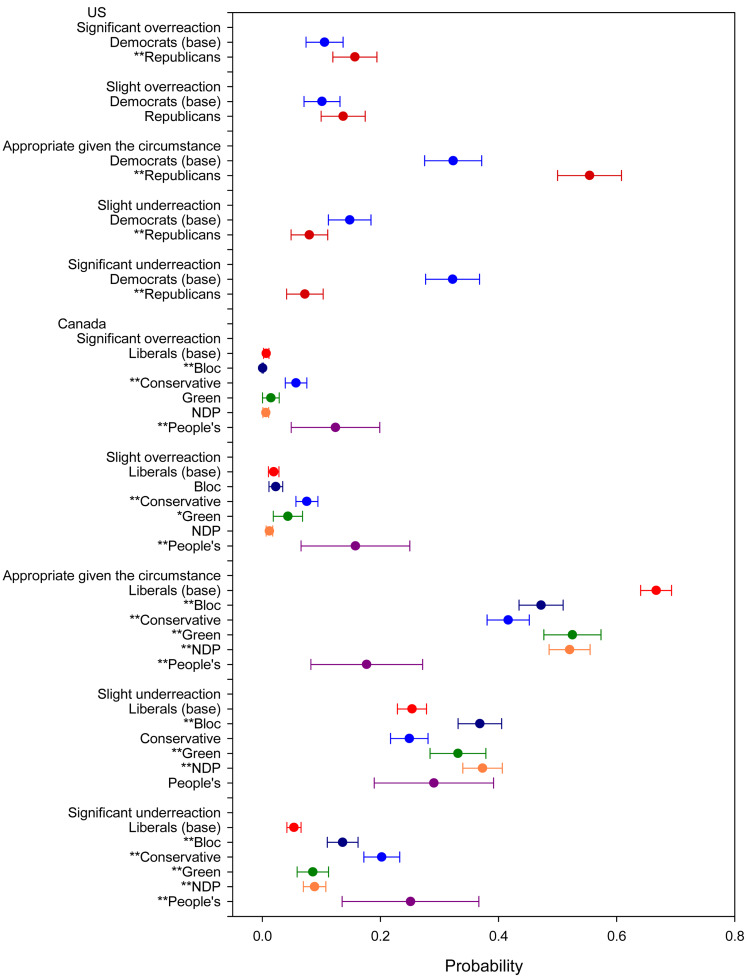
Government reaction. Note: *significant at 0.1 level; **significant at 0.05 level.

With respect to assessments of the *Government Reaction* (Figure 2), Democrats are significantly more likely than Republicans to say it was a slight or significant underreaction (47% versus 15%). On this overtly political assessment, there are also statistically significant differences between Liberals and supporters of all other parties in Canada. Of Liberals, 67 per cent said the response was appropriate. Only 18 per cent of PPC supporters, 37 per cent of Bloc supporters, 42 per cent of Conservatives, 52 per cent of NDP supporters, and 53 per cent of Green supporters said the same. Bloc, Green and NDP supporters were more likely to say it was an underreaction. Interestingly, while both Conservative and PPC supporters were more likely to declare the government response an underreaction, they were also more likely to say it was an overreaction. Conservative and PPC supporters are far from united in their assessment of the federal government's reaction to COVID-19 except that they agree it was not appropriate.

**Figure 3.**
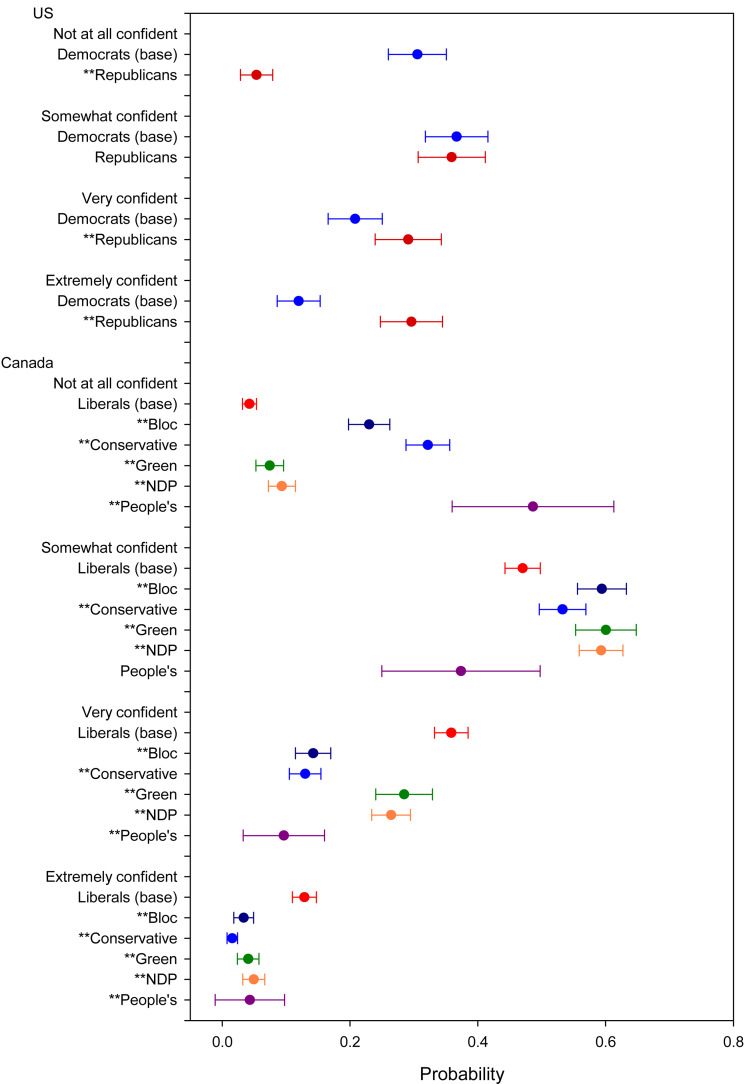
Government confidence. Note: *significant at 0.1 level; **significant at 0.05 level.

Turning to *Government Confidence* (Figure 3), it is unsurprising that Democrats are less likely than Republicans to say that they are very or extremely confident in the federal government's ability to safeguard the health and well-being of the American people during the pandemic (33% versus 69%). In Canada, 49 per cent of Liberals said that they are very or extremely confident in the federal government. Again, not surprisingly, confidence is lower amongst supporters of the other parties: Green, 33 per cent; NDP, 31 per cent; Bloc, 18 per cent; Conservatives, 15 per cent; and PPC, 14 per cent. Each of these differences is statistically significant.

**Figure 4.**
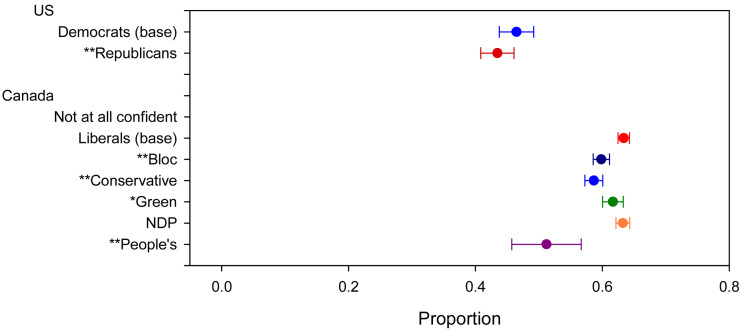
COVID behaviours. Note: *significant at 0.1 level; **significant at 0.05 level.

Next, we look at partisan differences in the self-reported behavioural responses to COVID-19 (Figure 4). On average, Democrats have made 46 per cent of the 22 behavioural changes listed in the survey question. In contrast, Republicans have on average made 43 per cent of these changes. This is not a large difference, but it is statistically significant. Liberals in Canada have made 63 per cent of the behavioural changes, on average. This is substantially greater than Republicans or Democrats in the US. The behaviours of Green and NDP supporters in Canada are indistinguishable from Liberals. Conservatives, PPC and Bloc supporters are different from Liberals in terms of their reported behaviours, with each having made fewer changes to their daily routines. Bloc supporters have made 60 per cent, Conservatives 59 per cent and PPC supporters 51 per cent of the changes. The greatest behavioural differences are between Liberal and PPC supporters, although even PPC supporters have engaged in great levels of compliance with public health guidance than Democrats in the US. The next greatest differences are between Democrats and Republicans, and between Liberals and Bloc or Conservative supporters. Each of these partisan differences is statistically significant. However, with the exception of PPC supporters, of which there are few, these behavioural differences are small.

## Conclusion

Although we did not expect to find large partisan differences in Canada, because of the documented elite consensus among major parties, partisanship seems to drive political assessments of the federal government's response to COVID-19 in both Canada and the US. The gap between Democrats and Republicans in their confidence in the government to handle the crisis is the same as the gap between Liberals and supporters of the Conservatives and PPC. It is more surprising that there are partisan differences in concern about COVID-19—not just in the US but also in Canada. Despite consensus of Canadian political elites about the dangers of COVID-19 and the necessity to take serious actions to combat it, partisanship still affects political assessments related to COVID. It seems that partisanship may play a role in voter assessments of a government's policy and related messaging even when that policy is not explicitly politicized.

Encouragingly, we find that these differences are much smaller when it comes to behavioural responses to the pandemic. The gap between Republicans and Democrats, and between Liberals and supporters of most other parties in Canada, is very small in magnitude. The largest difference is between the small number of Peoples’ Party supporters and Liberals. This suggests that while overtly political assessments are partisan, this polarization is dampened down when it comes to actual behavioural responses to the pandemic. Partisan divisions have the potential to pose public health risks by reducing healthy behavioural responses to COVID-19, but that potential is not being realized in a substantively significant way in either the US or Canada.

## Notes

1 Canadian respondents are asked how concerned they were about a range of COVID-19 items, while Americans were just asked if they were concerned about the pandemic generally. For the Canadian respondents, we use the maximum concern expressed among the first three items. Each is about them or others contracting COVID-19.

2 Respondents were provided a list of 22 items and could select all that applied.

3 We ran a multinomial, rather than ordered model, because we do not believe all respondents treated the response scale for *Government Reaction* as ordered. The multinomial model does not assume the response options are ordered, but it also does not assume they are not, so the disadvantage of using a multinomial model is that it may be less efficient and result in larger standard errors. As a robustness test, we re-ran the analysis using ordered probit models (see the Supplementary Materials, available online).

4 Ideology was not included because, to the extent that partisan identity/support determines ideology, including ideology in our models would result in an observational data form of post-treatment bias. That said, the results do not differ much when controlling for ideology. The only notable difference is that partisan differences in COVID behaviours in the US are no longer statistically significant, although the magnitude does not change.

5 All tests of statistical significance are 0.05 two-tailed tests.

## Supplementary material

For supplementary material accompanying this paper visit http://dx.doi.org/10.1017/S0008423920000463. click here to view supplementary material
