# Supplementary material for: Novel Coronavirus, Old Partisanship: COVID-19 Attitudes and Behaviours in the United States and Canada
Source: Can J Polit Sci. 2020 May 12:1–8. doi: 10.1017/S0008423920000463 (PMC7256215; doi:10.1017/S0008423920000463)
Supplement: Supplementary file 1 [file S0008423920000463sup001.docx]

**Online Supplementary Material for
*Novel coronavirus, old partisanship: COVID-19 attitudes and behaviors***

***in the United States and Canada****by
Mark Pickup, Dominik Stecuła and Clifton van der Linden*

Sampling – US

The American survey was fielded through Lucid on March 31, 2020 ($n=$ 1,009). Previous research has found that Lucid is a high-quality source of public opinion data (Coppock & McClellan 2019) and through basic demographic quotas tends to produce samples broadly representative of the US population. We further generate weighting variable using the iterative proportional fitting algorithm based on whether the respondent is Hispanic or not, white or not, and educational attainment. We use benchmark data from the US Census’s Current Population Survey (CPS) from February of 2018.

Coppock, A., & McClellan, O.A. (2019). Validating the demographic, political, psychological, and experimental results obtained from a new source of online survey respondents. *Research & Politics*, *6*(1), <https://doi.org/10.1177/2053168018822174>

Sampling – Canada

The Canadian data comes from Vox Pop Labs COVID-19 Monitor initiative, employing data from a rolling sample, collected between 20 March and 7 April 2020 ($n=$ 9,889). The sample was drawn from an online panel of approximately 650,000 individuals residing in Canada. Weights benchmarked on the 2016 Canadian Census are based on age group, sex, highest level of educational attainment, vote recall in the 2019 Canadian federal election, and region.

Survey Questions

*COVID-19 Concern – US*

How concerned are you about the coronavirus pandemic?

Not at all concerned

A little concerned

Somewhat concerned

Very concerned

*COVID-19 Concern – Canada*

How concerned would you say you are right now about each of the following?

Personally contracting COVID-19

A friend or family member contracting COVID-19

Someone in your local community contracting COVID-19

Your ability to pay your bills

Your job security

The availability of food and supplies in your area

The ability of the public health care system to provide adequate care in the event of illness or injury

The Canadian economy

The global economy

The government response to the COVID-19 pandemic

Other (Please specify):

Not at all concerned

A little concerned

Somewhat concerned

Very concerned

*COVID-19 Behaviours – Canada and US*

What changes, if any, have you made to your normal routine in response to the COVID-19 pandemic? (Please select all that apply)

Washing hands more often with soap and water

Cleaning and disinfecting frequently touched surfaces and objects more often

Using alcohol-based hand sanitizers more often

Not going into work or working from home more than usual

Avoiding all non-essential in-person contact outside your household

Staying in a separate room from others in your home

Using a separate bathroom from others in your home

Avoiding gatherings of more than 10 people

Avoiding gatherings of more than 50 people

Avoiding common greetings, such as handshakes

Avoiding crowded places

Keeping a distance of at least 2 arms lengths (approximately 6 ft) from others, as much as possible

Coughing or sneezing into a tissue or the bend of your arm instead of your hand

Avoiding touching your eyes, nose, or mouth with unwashed hands

Avoiding travel outside America

Avoiding travel within America

Shifting more of your shopping from physical stores to online

Avoiding people who are more likely to have come in contact with the coronavirus such as those who have recently traveled internationally

Avoiding public transit

Wearing a disposable mask

Wearing disposable gloves

Other

*Government Response – Canada and US*

Do you believe that the present action being taken by the federal government in response to the coronavirus (COVID-19) is an overreaction, an underreaction, or appropriate given the circumstances?

Significant overreaction

Slight overreaction

Appropriate given the circumstances

Slight underreaction

Significant underreaction

Don’t know

*Government Confidence – Canada and US*

How confident are you in the federal government’s ability to safeguard the health and well-being of the American people during the coronavirus (COVID-19) pandemic?

Not at all confident

Somewhat confident

Very confident

Extremely confident

Don’t know

*Partisan identity – US*

Generally speaking, do you usually think of yourself as a Republican, a Democrat, an Independent, or something else?

- Republican
- Democrat
- Independent
- None of these

Table SI.1 COVID Concern, US

|  | Not at all concerned | A little concerned | Somewhat concerned | Very concerned  (Base Category) |
| --- | --- | --- | --- | --- |
| Democrats (Base Category) |  |  |  |  |
|  |  |  |  |  |
| Republican | 0.522 | 0.546 | 0.089 |  |
|  | (0.236)* | (0.151)** | (0.113) |  |
| Age | -0.002 | -0.027 | -0.001 |  |
|  | (0.012) | (0.009)** | (0.006) |  |
| Gender | -1.401 | -0.784 | -0.380 |  |
|  | (0.517)** | (0.265)** | (0.209) |  |
| Under $15,000 (Base Category) |  |  |  |  |
|  |  |  |  |  |
| $15,000 to $19,999 | -0.575 | -0.482 | 0.125 |  |
|  | (0.796) | (0.601) | (0.422) |  |
| $20,000 to $24,999 | -0.549 | -1.055 | -0.360 |  |
|  | (0.784) | (0.516)* | (0.454) |  |
| $25,000 to $29,999 | 0.019 | -0.687 | 0.788 |  |
|  | (0.785) | (0.639) | (0.461) |  |
| $30,000 to $34,999 | 0.221 | 0.368 | 1.137 |  |
|  | (0.981) | (0.645) | (0.527)* |  |
| $35,000 to $39,999 | 0.119 | 0.006 | 0.478 |  |
|  | (0.753) | (0.509) | (0.447) |  |
| $40,000 to $44,999 | -0.220 | -0.055 | 0.853 |  |
|  | (1.296) | (0.672) | (0.582) |  |
| $45,000 to $49,999 | -0.268 | 0.031 | 0.304 |  |
|  | (0.994) | (0.684) | (0.559) |  |
| $50,000 to $54,999 | -15.797 | 0.934 | 1.195 |  |
|  | (0.647)** | (0.558) | (0.529)* |  |
| $55,000 to $59,999 | 0.660 | -1.830 | 0.690 |  |
|  | (0.898) | (1.063) | (0.585) |  |
| $60,000 to $64,999 | -15.593 | -0.191 | 1.243 |  |
|  | (0.639)** | (0.921) | (0.582)* |  |
| $65,000 to $69,999 | -16.679 | -0.796 | 0.117 |  |
|  | (0.639)** | (0.846) | (0.634) |  |
| $70,000 to $74,999 | -0.147 | -2.064 | -0.330 |  |
|  | (1.066) | (1.177) | (0.651) |  |
| $75,000 to $79,999 | -0.633 | -1.034 | -0.004 |  |
|  | (1.345) | (0.645) | (0.557) |  |
| $80,000 to $84,999 | -15.783 | -0.236 | -30.596 |  |
|  | (1.064)** | (1.344) | (1.624)** |  |
| $85,000 to $89,999 | -17.065 | -0.264 | -0.191 |  |
|  | (0.950)** | (0.853) | (0.903) |  |
| $90,000 to $94,999 | -16.107 | -1.110 | 0.012 |  |
|  | (0.787)** | (1.000) | (0.726) |  |
| $95,000 to $99,999 | -1.173 | 0.116 | -0.108 |  |
|  | (1.172) | (0.605) | (0.647) |  |
| $100,000 to $124,999 | -16.712 | -16.888 | -0.100 |  |
|  | (0.790)** | (0.519)** | (0.526) |  |
| $125,000 to $149,999 | -0.326 | -1.452 | -1.794 |  |
|  | (1.113) | (0.811) | (1.141) |  |
| $150,000 to $174,999 | -1.385 | -2.531 | -0.702 |  |
|  | (1.289) | (1.124)* | (0.686) |  |
| $175,000 to $199,999 | -16.320 | -16.717 | -0.009 |  |
|  | (1.036)** | (0.646)** | (0.916) |  |
| $200,000 to $249,999 | -16.800 | -17.066 | -0.634 |  |
|  | (1.178)** | (0.815)** | (0.989) |  |
| $250,000 and above | -16.260 | -16.648 | 0.383 |  |
|  | (0.949)** | (0.668)** | (0.826) |  |

Table SI.1 COVID Concern, US (cont.)

|  | Not at all concerned | A little concerned | Somewhat concerned | Very concerned  (Base Category) |
| --- | --- | --- | --- | --- |
| White (Base Category) |  |  |  |  |
|  |  |  |  |  |
| Black or African-American | 0.606 | 0.153 | -0.519 |  |
|  | (0.585) | (0.377) | (0.330) |  |
| American Indian or Alaska native | 2.011 | 1.240 | 1.628 |  |
|  | (1.160) | (1.064) | (0.912) |  |
| Asian: Asian Indian | -15.687 | -1.877 | -0.612 |  |
|  | (0.780)** | (1.030) | (0.648) |  |
| Asian: Chinese | -13.679 | 1.059 | 0.633 |  |
|  | (1.386)** | (1.389) | (1.106) |  |
| Asian: Filipino | -17.166 | -16.410 | -18.192 |  |
|  | (1.517)** | (1.284)** | (1.113)** |  |
| Asian: Japanese | -0.711 | -15.629 | 15.238 |  |
|  | (0.976) | (1.695)** | (1.192)** |  |
| Asian: Vietnamese | -18.208 | -17.926 | -17.536 |  |
|  | (1.755)** | (1.330)** | (1.210)** |  |
| Asian: other | -15.967 | -18.334 | -18.466 |  |
|  | (1.321)** | (1.169)** | (0.900)** |  |
| Pacific Islander: native Hawaiian | -17.193 | -17.784 | -17.422 |  |
|  | (1.431)** | (1.117)** | (1.079)** |  |
| Some other race | -15.976 | -1.019 | -0.588 |  |
|  | (0.410)** | (0.555) | (0.330) |  |
| Some high school (Base Category) |  |  |  |  |
|  |  |  |  |  |
| High school graduate | -0.124 | -0.526 | -0.383 |  |
|  | (0.944) | (0.713) | (0.850) |  |
| Post-high school vocational training | -15.816 | -0.206 | -0.863 |  |
|  | (1.149)** | (1.186) | (1.185) |  |
| Completed some college but no degree | 0.303 | -0.420 | -0.181 |  |
|  | (1.051) | (0.742) | (0.865) |  |
| Associates degree | -0.347 | -1.137 | -1.023 |  |
|  | (1.258) | (0.889) | (0.967) |  |
| Bachelor’s degree | -0.128 | -0.561 | -0.396 |  |
|  | (0.970) | (0.724) | (0.857) |  |
| Masters or professional degree | -0.798 | -0.780 | -0.698 |  |
|  | (1.258) | (0.884) | (0.902) |  |
| Doctorate degree | -1.799 | -18.037 | -18.120 |  |
|  | (1.308) | (1.360)** | (1.238)** |  |
| Northeast (Base Category) |  |  |  |  |
|  |  |  |  |  |
| Midwest | 0.791 | -0.265 | -0.401 |  |
|  | (0.602) | (0.425) | (0.316) |  |
| South | 0.364 | 0.133 | -0.194 |  |
|  | (0.580) | (0.369) | (0.262) |  |
| West | -0.056 | 0.603 | 0.221 |  |
|  | (0.822) | (0.406) | (0.300) |  |
| Constant | -0.999 | 1.009 | 0.235 |  |
|  | (1.325) | (0.972) | (0.914) |  |
| *N* | 690 |  |  |  |

* *p*<0.05; ** *p*<0.01

Table SI.2 COVID Concern, Canada

|  | Not at all concerned | Slightly concerned | Somewhat concerned | Very concerned  (Base Category) |
| --- | --- | --- | --- | --- |
| Bloc | 0.050 | 0.236 | 0.109 |  |
|  | (0.260) | (0.152) | (0.138) |  |
| Conservative | 0.443 | 0.388 | 0.048 |  |
|  | (0.193)* | (0.138)** | (0.116) |  |
| Green | 0.020 | -0.084 | 0.205 |  |
|  | (0.261) | (0.162) | (0.135) |  |
| Liberals (Base Category) |  |  |  |  |
|  |  |  |  |  |
| NDP | -0.360 | -0.161 | 0.110 |  |
|  | (0.211) | (0.138) | (0.108) |  |
| PPC | 1.004 | 0.569 | 0.565 |  |
|  | (0.449)* | (0.320) | (0.273)* |  |
| age | -0.184 | 0.127 | 0.114 |  |
|  | (0.086)* | (0.061)* | (0.049)* |  |
| gender | -0.290 | -0.497 | -0.402 |  |
|  | (0.147)* | (0.103)** | (0.084)** |  |
| Income: 0 to 50K (Base Category) |  |  |  |  |
|  |  |  |  |  |
| Income: 50 to 1000K | -0.144 | 0.038 | 0.064 |  |
|  | (0.163) | (0.113) | (0.090) |  |
| Income: 100K+ | -0.503 | -0.031 | 0.010 |  |
|  | (0.171)** | (0.122) | (0.096) |  |
| English (Base Category) |  |  |  |  |
|  |  |  |  |  |
| French | 0.278 | 0.365 | 0.121 |  |
|  | (0.341) | (0.236) | (0.180) |  |
| Other | 0.578 | 0.021 | 0.083 |  |
|  | (0.258)* | (0.213) | (0.161) |  |
| High school or below (Base Category) |  |  |  |  |
|  |  |  |  |  |
| College or trade school | -0.436 | -0.392 | -0.231 |  |
|  | (0.184)* | (0.134)** | (0.114)* |  |
| University education | -0.703 | -0.206 | -0.169 |  |
|  | (0.170)** | (0.122) | (0.103) |  |
| Alberta (Base Category) |  |  |  |  |
|  |  |  |  |  |
| Atlantic | 0.264 | 0.314 | -0.085 |  |
|  | (0.361) | (0.278) | (0.215) |  |
| BC | -0.200 | -0.529 | 0.003 |  |
|  | (0.322) | (0.227)* | (0.169) |  |
| Manitoba/Saskatchewan | -0.029 | -0.247 | 0.107 |  |
|  | (0.404) | (0.284) | (0.215) |  |
| Ontario | 0.082 | -0.261 | -0.069 |  |
|  | (0.255) | (0.184) | (0.144) |  |
| Quebec | -0.171 | 0.280 | 0.223 |  |
|  | (0.404) | (0.285) | (0.213) |  |
| Constant | -0.483 | -0.404 | -0.012 |  |
|  | (0.410) | (0.323) | (0.258) |  |
| *N* | 9,058 |  |  |  |

* *p*<0.05; ** *p*<0.01

Table SI.3 Government Response, US

|  | Significant  overreaction | Slight  overreaction | Appropriate  (Base Category) | Slight  under reaction | Significant  under reaction |
| --- | --- | --- | --- | --- | --- |
| Democrats (Base) |  |  |  |  |  |
|  |  |  |  |  |  |
| Republican | -0.037 | -0.038 |  | -0.692 | -1.284 |
|  | (0.173) | (0.154) |  | (0.166)** | (0.174)** |
| Age | -0.032 | -0.044 |  | -0.006 | -0.002 |
|  | (0.010)** | (0.010)** |  | (0.010) | (0.008) |
| Gender | -0.729 | -0.665 |  | -0.281 | 0.020 |
|  | (0.323)* | (0.316)* |  | (0.304) | (0.255) |
| Under $15,000 (Base) |  |  |  |  |  |
|  |  |  |  |  |  |
| $15,000 to $19,999 | -0.676 | 0.434 |  | -0.428 | -0.095 |
|  | (0.822) | (0.673) |  | (0.634) | (0.565) |
| $20,000 to $24,999 | -0.385 | -0.217 |  | -0.162 | 0.280 |
|  | (0.669) | (0.584) |  | (0.618) | (0.570) |
| $25,000 to $29,999 | -0.566 | -0.496 |  | -0.390 | 0.194 |
|  | (0.678) | (0.713) |  | (0.676) | (0.528) |
| $30,000 to $34,999 | 0.627 | 0.548 |  | 0.192 | 0.611 |
|  | (0.671) | (0.755) |  | (0.736) | (0.597) |
| $35,000 to $39,999 | -0.851 | 0.894 |  | 0.151 | 0.360 |
|  | (0.867) | (0.572) |  | (0.599) | (0.551) |
| $40,000 to $44,999 | -0.509 | 0.242 |  | -0.034 | 0.523 |
|  | (0.889) | (0.906) |  | (0.767) | (0.679) |
| $45,000 to $49,999 | -15.601 | 0.447 |  | 1.522 | 1.230 |
|  | (0.618)** | (0.952) |  | (0.751)* | (0.828) |
| $50,000 to $54,999 | -0.174 | 0.344 |  | -0.039 | 0.426 |
|  | (0.818) | (0.655) |  | (0.660) | (0.575) |
| $55,000 to $59,999 | -0.312 | 0.977 |  | 0.863 | 0.863 |
|  | (1.217) | (0.744) |  | (0.829) | (0.747) |
| $60,000 to $64,999 | -0.741 | -0.027 |  | -0.449 | -0.625 |
|  | (1.092) | (0.954) |  | (0.866) | (0.731) |
| $65,000 to $69,999 | -0.674 | -0.202 |  | 1.049 | -0.880 |
|  | (1.151) | (1.224) |  | (0.906) | (1.021) |
| $70,000 to $74,999 | 0.772 | -16.029 |  | -0.587 | 0.183 |
|  | (0.698) | (0.581)** |  | (1.145) | (0.717) |
| $75,000 to $79,999 | 0.397 | -0.332 |  | -0.417 | 0.544 |
|  | (0.786) | (0.828) |  | (0.877) | (0.688) |
| $80,000 to $84,999 | 18.310 | 17.110 |  | 15.897 | 16.920 |
|  | (0.878)** | (1.422)** |  | (0.723)** | (0.776)** |
| $85,000 to $89,999 | -16.923 | -1.497 |  | -1.188 | -1.749 |
|  | (0.631)** | (1.218) |  | (1.084) | (1.185) |
| $90,000 to $94,999 | -1.387 | 0.939 |  | -16.364 | -0.597 |
|  | (1.164) | (0.774) |  | (0.559)** | (1.037) |
| $95,000 to $99,999 | 1.304 | -0.138 |  | 1.450 | 0.347 |
|  | (0.807) | (1.236) |  | (0.864) | (0.842) |
| $100,000 to $124,999 | -0.801 | -0.581 |  | -0.077 | -0.089 |
|  | (0.800) | (0.783) |  | (0.704) | (0.678) |
| $125,000 to $149,999 | 0.764 | 0.327 |  | -0.694 | 0.616 |
|  | (0.795) | (1.017) |  | (1.127) | (0.717) |
| $150,000 to $174,999 | 0.695 | -0.460 |  | 0.095 | 1.364 |
|  | (0.733) | (0.987) |  | (0.931) | (0.766) |
| $175,000 to $199,999 | 0.910 | -16.487 |  | 0.138 | 0.196 |
|  | (1.107) | (0.924)** |  | (1.218) | (1.299) |
| $200,000 to $249,999 | 1.038 | 0.874 |  | -15.368 | 2.361 |
|  | (1.078) | (1.502) |  | (2.345)** | (1.835) |
| $250,000 and above | 0.496 | 0.139 |  | -16.028 | 0.928 |
|  | (1.130) | (1.423) |  | (0.733)** | (0.940) |

Table SI.3 Government Response, US (cont.)

|  | Significant  overreaction | Slight  overreaction | Appropriate  (Base Category) | Slight  under reaction | Significant  under reaction |
| --- | --- | --- | --- | --- | --- |
| White (Base Category) |  |  |  |  |  |
|  |  |  |  |  |  |
| Black or African-American | -0.426 | -0.254 |  | -1.037 | -0.861 |
|  | (0.471) | (0.441) |  | (0.436)* | (0.373)* |
| American Indian or Alaska native | -14.731 | -15.510 |  | 1.020 | 2.855 |
|  | (0.780)** | (0.726)** |  | (1.079) | (0.881)** |
| Asian: Asian Indian | 0.340 | 0.413 |  | -0.594 | -0.510 |
|  | (0.714) | (0.914) |  | (0.908) | (0.769) |
| Asian: Chinese | -17.017 | -17.339 |  | 0.363 | -0.923 |
|  | (1.757)** | (1.452)** |  | (1.057) | (2.627) |
| Asian: Filipino | 1.301 | 1.472 |  | -0.571 | 18.782 |
|  | (0.902) | (0.798) |  | (0.774) | (1.174)** |
| Asian: Japanese | -3.542 | 17.065 |  | 18.050 | -3.091 |
|  | (1.116)** | (1.474)** |  | (1.146)** | (1.573)* |
| Asian: Vietnamese | 22.023 | -1.374 |  | 0.731 | 1.122 |
|  | (1.391)** | (1.152) |  | (0.875) | (0.796) |
| Asian: other | 23.050 | -0.014 |  | -0.564 | -1.123 |
|  | (1.297)** | (0.714) |  | (0.656) | (0.547)* |
| Pacific Islander: native Hawaiian | 22.688 | -0.133 |  | -1.118 | -0.609 |
|  | (1.186)** | (0.636) |  | (0.557)* | (0.512) |
| Some other race | 0.567 | -0.304 |  | -0.188 | -0.377 |
|  | (0.499) | (0.483) |  | (0.494) | (0.444) |
| Some high school (Base Category) | 0.000 | 0.000 |  | 0.000 | 0.000 |
|  | (0.000) | (0.000) |  | (0.000) | (0.000) |
| High school graduate | -0.364 | 1.036 |  | -0.236 | 0.098 |
|  | (0.911) | (1.016) |  | (1.029) | (1.300) |
| Post-high school vocational training | -15.593 | -14.411 |  | -16.656 | 0.197 |
|  | (1.164)** | (1.221)** |  | (1.226)** | (1.668) |
| Completed some college but no degree | 0.433 | 1.556 |  | -0.176 | 0.555 |
|  | (0.960) | (1.074) |  | (1.065) | (1.313) |
| Associates degree | -0.903 | 1.562 |  | -0.841 | 1.084 |
|  | (1.257) | (1.252) |  | (1.263) | (1.392) |
| Bachelor’s degree | 0.215 | 1.874 |  | -0.146 | 0.440 |
|  | (0.930) | (1.037) |  | (1.035) | (1.301) |
| Masters or professional degree | 1.127 | 2.183 |  | -0.179 | 0.551 |
|  | (1.010) | (1.169) |  | (1.112) | (1.348) |
| Doctorate degree | 18.396 | 19.878 |  | -0.214 | -0.399 |
|  | (1.560)** | (1.834)** |  | (1.470) | (1.594) |
| Northeast (Base Category) |  |  |  |  |  |
|  |  |  |  |  |  |
| Midwest | -0.312 | -0.231 |  | -0.322 | -0.139 |
|  | (0.479) | (0.451) |  | (0.481) | (0.385) |
| South | -0.061 | -0.488 |  | -0.019 | -0.256 |
|  | (0.360) | (0.386) |  | (0.418) | (0.338) |
| West | -0.427 | -0.687 |  | 0.120 | -0.332 |
|  | (0.504) | (0.468) |  | (0.447) | (0.394) |
| Constant | 1.334 | 0.448 |  | 1.153 | 1.180 |
|  | (1.143) | (1.319) |  | (1.148) | (1.328) |
| *N* | 667 |  |  |  |  |

Table SI.4 Government Response, Canada

|  | Significant  overreaction | Slight  overreaction | Appropriate  (Base Category) | Slight  under reaction | Significant  under reaction |
| --- | --- | --- | --- | --- | --- |
| Bloc | -1.264 | 0.811 |  | 0.853 | 1.665 |
|  | (0.941) | (0.397)* |  | (0.131)** | (0.215)** |
| Conservative | 2.665 | 1.717 |  | 0.489 | 1.820 |
|  | (0.413)** | (0.270)** |  | (0.121)** | (0.173)** |
| Green | 0.768 | 0.994 |  | 0.464 | 0.716 |
|  | (0.637) | (0.389)* |  | (0.137)** | (0.222)** |
| Liberals (Base Category) | 0.000 | 0.000 |  | 0.000 | 0.000 |
|  | (0.000) | (0.000) |  | (0.000) | (0.000) |
| NDP | -0.032 | -0.317 |  | 0.530 | 0.754 |
|  | (0.569) | (0.364) |  | (0.108)** | (0.183)** |
| PPC | 4.033 | 3.240 |  | 1.426 | 2.806 |
|  | (0.585)** | (0.506)** |  | (0.369)** | (0.444)** |
| age | -0.201 | -0.189 |  | -0.268 | -0.106 |
|  | (0.165) | (0.117) |  | (0.051)** | (0.068) |
| gender | -1.101 | -0.532 |  | -0.098 | -0.338 |
|  | (0.334)** | (0.213)* |  | (0.083) | (0.131)** |
| Income: 0 to 50K (Base Category) | 0.000 | 0.000 |  | 0.000 | 0.000 |
|  | (0.000) | (0.000) |  | (0.000) | (0.000) |
| Income: 50 to 1000K | -0.655 | 0.118 |  | -0.142 | -0.067 |
|  | (0.323)* | (0.234) |  | (0.089) | (0.147) |
| Income: 100K+ | -0.494 | 0.072 |  | -0.164 | -0.119 |
|  | (0.316) | (0.243) |  | (0.096) | (0.152) |
| English (Base Category) | 0.000 | 0.000 |  | 0.000 | 0.000 |
|  | (0.000) | (0.000) |  | (0.000) | (0.000) |
| French | -0.119 | -0.587 |  | -0.290 | 0.006 |
|  | (0.638) | (0.560) |  | (0.191) | (0.304) |
| Other | -0.060 | 0.174 |  | 0.346 | 0.215 |
|  | (0.416) | (0.462) |  | (0.158)* | (0.242) |
| High school or below (Base Category) | 0.000 | 0.000 |  | 0.000 | 0.000 |
|  | (0.000) | (0.000) |  | (0.000) | (0.000) |
| College or trade school | -0.372 | -0.184 |  | 0.145 | 0.389 |
|  | (0.366) | (0.247) |  | (0.111) | (0.166)* |
| University education | -0.380 | -0.571 |  | -0.181 | -0.112 |
|  | (0.318) | (0.239)* |  | (0.101) | (0.157) |
| Alberta (Base Category) | 0.000 | 0.000 |  | 0.000 | 0.000 |
|  | (0.000) | (0.000) |  | (0.000) | (0.000) |
| Atlantic | 0.818 | -0.578 |  | 0.034 | -0.048 |
|  | (0.695) | (0.488) |  | (0.232) | (0.375) |
| BC | 0.731 | 0.060 |  | 0.100 | 0.495 |
|  | (0.503) | (0.364) |  | (0.176) | (0.256) |
| Manitoba/Saskatchewan | -0.657 | -0.628 |  | 0.246 | 0.191 |
|  | (0.757) | (0.488) |  | (0.213) | (0.367) |
| Ontario | 0.422 | -0.174 |  | 0.103 | 0.625 |
|  | (0.437) | (0.304) |  | (0.149) | (0.219)** |
| Quebec | -0.463 | 0.068 |  | 0.300 | -0.024 |
|  | (0.772) | (0.601) |  | (0.227) | (0.351) |
| Constant | -2.332 | -1.818 |  | -0.168 | -2.200 |
|  | (0.772)** | (0.653)** |  | (0.246) | (0.395)** |
| *N* | 8,925 |  |  |  |  |

Table SI.5 Government Confidence, US

|  | Not at all confident | Somewhat confident  (Base Category) | Very confident | Extremely confident |
| --- | --- | --- | --- | --- |
| Democrats (Base Category) |  |  |  |  |
|  |  |  |  |  |
| Republican | -1.043 |  | 0.226 | 0.438 |
|  | (0.178)** |  | (0.128) | (0.138)** |
| Age | 0.015 |  | -0.012 | -0.014 |
|  | (0.009) |  | (0.007) | (0.008) |
| Gender | 0.266 |  | 0.008 | -0.255 |
|  | (0.250) |  | (0.235) | (0.261) |
| Under $15,000 (Base Category) |  |  |  |  |
|  |  |  |  |  |
| $15,000 to $19,999 | -1.164 |  | -0.739 | -0.419 |
|  | (0.565)* |  | (0.539) | (0.527) |
| $20,000 to $24,999 | -0.188 |  | 0.029 | -0.170 |
|  | (0.542) |  | (0.453) | (0.555) |
| $25,000 to $29,999 | -0.370 |  | 0.030 | 0.185 |
|  | (0.541) |  | (0.526) | (0.529) |
| $30,000 to $34,999 | -0.756 |  | -1.707 | -16.962 |
|  | (0.575) |  | (0.747)* | (0.413)** |
| $35,000 to $39,999 | -0.203 |  | 0.020 | -0.281 |
|  | (0.552) |  | (0.521) | (0.555) |
| $40,000 to $44,999 | -1.826 |  | -0.472 | -1.761 |
|  | (0.846)* |  | (0.607) | (0.865)* |
| $45,000 to $49,999 | -1.008 |  | -1.097 | -1.071 |
|  | (0.656) |  | (0.693) | (0.740) |
| $50,000 to $54,999 | -0.054 |  | -0.261 | -0.108 |
|  | (0.574) |  | (0.555) | (0.640) |
| $55,000 to $59,999 | -0.314 |  | -0.262 | -2.097 |
|  | (0.683) |  | (0.676) | (1.053)* |
| $60,000 to $64,999 | -0.335 |  | 0.061 | -0.075 |
|  | (0.745) |  | (0.682) | (0.812) |
| $65,000 to $69,999 | -16.839 |  | -0.506 | -1.076 |
|  | (0.594)** |  | (0.693) | (0.723) |
| $70,000 to $74,999 | 0.924 |  | 0.874 | 0.784 |
|  | (0.852) |  | (0.765) | (0.814) |
| $75,000 to $79,999 | -0.307 |  | 0.640 | 0.160 |
|  | (0.781) |  | (0.618) | (0.653) |
| $80,000 to $84,999 | 0.537 |  | 0.790 | 0.388 |
|  | (1.381) |  | (1.582) | (1.277) |
| $85,000 to $89,999 | -17.040 |  | 0.033 | -1.358 |
|  | (0.663)** |  | (0.815) | (1.210) |
| $90,000 to $94,999 | 0.234 |  | 1.278 | 0.997 |
|  | (1.195) |  | (1.086) | (0.945) |
| $95,000 to $99,999 | -0.822 |  | -0.035 | -0.299 |
|  | (0.768) |  | (0.712) | (0.792) |
| $100,000 to $124,999 | 0.367 |  | 0.031 | 0.241 |
|  | (0.633) |  | (0.666) | (0.638) |
| $125,000 to $149,999 | 1.114 |  | 0.202 | 1.582 |
|  | (0.834) |  | (0.991) | (0.804)* |
| $150,000 to $174,999 | 0.576 |  | 0.672 | 0.328 |
|  | (0.852) |  | (0.716) | (0.657) |
| $175,000 to $199,999 | -0.443 |  | 0.026 | 1.173 |
|  | (1.206) |  | (1.703) | (1.285) |
| $200,000 to $249,999 | -0.914 |  | -0.861 | 0.245 |
|  | (1.399) |  | (0.947) | (1.123) |
| $250,000 and above | -0.191 |  | 1.195 | -0.740 |
|  | (1.321) |  | (1.004) | (1.256) |

Table SI.5 Government Confidence, US (cont.)

|  | Not at all confident | Somewhat confident  (Base Category) | Very confident | Extremely confident |
| --- | --- | --- | --- | --- |
| White (Base Category) |  |  |  |  |
|  |  |  |  |  |
| Black or African-American | -0.242 |  | 0.160 | 0.200 |
|  | (0.369) |  | (0.357) | (0.415) |
| American Indian or Alaska native | 2.496 |  | 0.756 | -16.101 |
|  | (0.923)** |  | (1.036) | (0.888)** |
| Asian: Asian Indian | -0.398 |  | 0.762 | -0.209 |
|  | (0.688) |  | (0.714) | (0.908) |
| Asian: Chinese | 0.130 |  | -16.821 | -0.963 |
|  | (1.194) |  | (0.919)** | (1.434) |
| Asian: Filipino | 19.492 |  | 0.700 | 0.411 |
|  | (1.174)** |  | (0.603) | (0.576) |
| Asian: Japanese | -18.889 |  | -17.707 | -16.171 |
|  | (1.680)** |  | (1.618)** | (1.657)** |
| Asian: Vietnamese | 2.889 |  | 0.963 | 21.224 |
|  | (0.949)** |  | (0.769) | (1.373)** |
| Asian: other | -18.790 |  | -17.962 | -18.452 |
|  | (1.143)** |  | (1.144)** | (1.151)** |
| Pacific Islander: native Hawaiian | -1.147 |  | 20.108 | 0.253 |
|  | (0.485)* |  | (1.110)** | (0.520) |
| Some other race | -0.277 |  | 0.029 | -0.195 |
|  | (0.403) |  | (0.398) | (0.440) |
| Some high school (Base Category) |  |  |  |  |
|  |  |  |  |  |
| High school graduate | 1.015 |  | 0.570 | 0.384 |
|  | (1.198) |  | (0.790) | (0.950) |
| Post-high school vocational training | 1.644 |  | 0.731 | 1.529 |
|  | (1.583) |  | (1.409) | (1.462) |
| Completed some college but no degree | 0.552 |  | -0.165 | -0.089 |
|  | (1.219) |  | (0.816) | (1.000) |
| Associates degree | 0.536 |  | 0.337 | 0.732 |
|  | (1.352) |  | (0.946) | (1.055) |
| Bachelor’s degree | 0.975 |  | 0.142 | 0.744 |
|  | (1.197) |  | (0.807) | (0.954) |
| Masters or professional degree | 1.467 |  | -0.266 | 1.019 |
|  | (1.252) |  | (0.932) | (1.020) |
| Doctorate degree | 1.270 |  | 18.285 | 18.026 |
|  | (1.519) |  | (1.419)** | (1.664)** |
| Northeast (Base Category) |  |  |  |  |
|  |  |  |  |  |
| Midwest | -0.191 |  | -0.413 | -0.008 |
|  | (0.378) |  | (0.363) | (0.382) |
| South | -0.024 |  | 0.116 | 0.072 |
|  | (0.331) |  | (0.307) | (0.336) |
| West | 0.321 |  | -0.213 | -0.389 |
|  | (0.360) |  | (0.372) | (0.439) |
| Constant | -0.681 |  | -0.409 | -0.777 |
|  | (1.315) |  | (0.926) | (1.174) |
| *N* | 670 |  |  |  |

Table SI.6 Government Confidence, Canada

|  | Not at all confident | Somewhat confident  (Base Category) | Very confident | Extremely confident |
| --- | --- | --- | --- | --- |
| Bloc | 1.475 |  | -0.850 | -1.446 |
|  | (0.203)** |  | (0.153)** | (0.302)** |
| Conservative | 1.835 |  | -1.175 | -2.351 |
|  | (0.169)** |  | (0.137)** | (0.300)** |
| Green | 0.291 |  | -0.536 | -1.425 |
|  | (0.223) |  | (0.136)** | (0.254)** |
| Liberals (Base Category) |  |  |  |  |
|  |  |  |  |  |
| NDP | 0.579 |  | -0.544 | -1.178 |
|  | (0.198)** |  | (0.108)** | (0.214)** |
| PPC | 2.571 |  | -1.149 | -0.974 |
|  | (0.326)** |  | (0.414)** | (0.713) |
| age | 0.094 |  | 0.016 | 0.131 |
|  | (0.068) |  | (0.051) | (0.078) |
| gender | -0.449 |  | -0.068 | -0.237 |
|  | (0.117)** |  | (0.087) | (0.149) |
| Income: 0 to 50K (Base Category) |  |  |  |  |
|  |  |  |  |  |
| Income: 50 to 1000K | -0.128 |  | 0.028 | 0.024 |
|  | (0.134) |  | (0.093) | (0.144) |
| Income: 100K+ | -0.073 |  | 0.001 | 0.056 |
|  | (0.136) |  | (0.099) | (0.155) |
| English (Base Category) |  |  |  |  |
|  |  |  |  |  |
| French | -0.004 |  | 0.203 | -0.402 |
|  | (0.274) |  | (0.238) | (0.323) |
| Other | -0.496 |  | -0.271 | -0.113 |
|  | (0.233)* |  | (0.166) | (0.219) |
| High school or below (Base Category) |  |  |  |  |
|  |  |  |  |  |
| College or trade school | 0.050 |  | -0.017 | -0.379 |
|  | (0.145) |  | (0.120) | (0.188)* |
| University education | -0.247 |  | 0.142 | -0.431 |
|  | (0.138) |  | (0.108) | (0.166)** |
| Alberta (Base Category) |  |  |  |  |
|  |  |  |  |  |
| Atlantic | 0.232 |  | 0.133 | 0.058 |
|  | (0.340) |  | (0.231) | (0.355) |
| BC | 0.401 |  | -0.126 | -0.212 |
|  | (0.226) |  | (0.181) | (0.288) |
| Manitoba/Saskatchewan | 0.240 |  | -0.303 | -0.516 |
|  | (0.302) |  | (0.217) | (0.418) |
| Ontario | 0.417 |  | -0.177 | -0.375 |
|  | (0.184)* |  | (0.158) | (0.245) |
| Quebec | 0.155 |  | -0.734 | -0.139 |
|  | (0.311) |  | (0.266)** | (0.381) |
| Constant | -2.110 |  | -0.034 | -0.671 |
|  | (0.353)** |  | (0.269) | (0.414) |
| *N* | 8,867 |  |  |  |

Table SI.7 COVID Behaviours, US

|  | Proportion of Behaviours |
| --- | --- |
| Democrats (Base Category) |  |
|  |  |
| Republican | -0.030 |
|  | (0.011)** |
| Age | 0.003 |
|  | (0.001)** |
| Gender | 0.054 |
|  | (0.021)* |
| Under $15,000 (Base Category) |  |
|  |  |
| $15,000 to $19,999 | 0.101 |
|  | (0.047)* |
| $20,000 to $24,999 | 0.111 |
|  | (0.048)* |
| $25,000 to $29,999 | 0.094 |
|  | (0.045)* |
| $30,000 to $34,999 | 0.077 |
|  | (0.049) |
| $35,000 to $39,999 | 0.076 |
|  | (0.045) |
| $40,000 to $44,999 | 0.058 |
|  | (0.068) |
| $45,000 to $49,999 | 0.083 |
|  | (0.061) |
| $50,000 to $54,999 | 0.117 |
|  | (0.056)* |
| $55,000 to $59,999 | 0.094 |
|  | (0.063) |
| $60,000 to $64,999 | 0.115 |
|  | (0.054)* |
| $65,000 to $69,999 | 0.174 |
|  | (0.057)** |
| $70,000 to $74,999 | 0.040 |
|  | (0.054) |
| $75,000 to $79,999 | 0.112 |
|  | (0.048)* |
| $80,000 to $84,999 | 0.015 |
|  | (0.048) |
| $85,000 to $89,999 | 0.109 |
|  | (0.077) |
| $90,000 to $94,999 | 0.184 |
|  | (0.068)** |
| $95,000 to $99,999 | 0.104 |
|  | (0.067) |
| $100,000 to $124,999 | 0.102 |
|  | (0.053) |
| $125,000 to $149,999 | 0.043 |
|  | (0.070) |
| $150,000 to $174,999 | 0.116 |
|  | (0.061) |
| $175,000 to $199,999 | 0.011 |
|  | (0.055) |
| $200,000 to $249,999 | -0.066 |
|  | (0.068) |
| $250,000 and above | 0.101 |
|  | (0.104) |

Table SI.7 COVID Behaviours, US (cont.)

|  | Proportion of Behaviours |
| --- | --- |
| White (Base Category) |  |
|  |  |
| Black or African-American | -0.039 |
|  | (0.034) |
| American Indian or Alaska native | 0.180 |
|  | (0.080)* |
| Asian: Asian Indian | 0.062 |
|  | (0.080) |
| Asian: Chinese | 0.175 |
|  | (0.138) |
| Asian: Filipino | 0.282 |
|  | (0.050)** |
| Asian: Japanese | 0.245 |
|  | (0.048)** |
| Asian: Vietnamese | -0.108 |
|  | (0.074) |
| Asian: other | 0.367 |
|  | (0.081)** |
| Pacific Islander: native Hawaiian | 0.366 |
|  | (0.043)** |
| Some other race | -0.018 |
|  | (0.037) |
| Some high school (Base Category) |  |
|  |  |
| High school graduate | 0.072 |
|  | (0.070) |
| Post-high school vocational training | 0.070 |
|  | (0.088) |
| Completed some college but no degree | 0.070 |
|  | (0.073) |
| Associates degree | 0.144 |
|  | (0.079) |
| Bachelor’s degree | 0.045 |
|  | (0.070) |
| Masters or professional degree | 0.064 |
|  | (0.076) |
| Doctorate degree | -0.148 |
|  | (0.166) |
| Northeast (Base Category) |  |
|  |  |
| Midwest | 0.035 |
|  | (0.033) |
| South | 0.007 |
|  | (0.029) |
| West | 0.010 |
|  | (0.031) |
| Constant | 0.131 |
|  | (0.088) |
| *R*^2^ | 0.16 |
| *N* | 690 |

* *p*<0.05; ** *p*<0.01

Table SI.8 COVID Behaviours, Canada

|  | Proportion of Behaviours |
| --- | --- |
| Bloc | -0.021 |
|  | (0.010)* |
| Conservative | -0.039 |
|  | (0.009)** |
| Green | -0.015 |
|  | (0.010) |
| Liberals (Base Category) |  |
|  |  |
| NDP | -0.000 |
|  | (0.008) |
| PPC | -0.107 |
|  | (0.028)** |
| age | -0.003 |
|  | (0.004) |
| gender | 0.047 |
|  | (0.006)** |
| Income: 0 to 50K |  |
|  |  |
| Income: 50 to 1000K | 0.004 |
|  | (0.007) |
| Income: 100K+ | 0.037 |
|  | (0.007)** |
| English (Base Category) |  |
|  |  |
| French | 0.013 |
|  | (0.013) |
| Other | 0.007 |
|  | (0.015) |
| High school or below (Base Category) |  |
|  |  |
| College or trade school | 0.026 |
|  | (0.009)** |
| University education | 0.029 |
|  | (0.008)** |
| Alberta (Base Category) |  |
|  |  |
| Atlantic | -0.005 |
|  | (0.018) |
| BC | 0.008 |
|  | (0.014) |
| Manitoba/Saskatchewan | -0.005 |
|  | (0.016) |
| Ontario | 0.002 |
|  | (0.012) |
| Quebec | -0.018 |
|  | (0.017) |
| Constant | 0.541 |
|  | (0.021)** |
| *R*^2^ | 0.05 |
| *N* | 9,082 |

* *p*<0.05; ** *p*<0.01

Ordered Probit Results

These results are largely similar to those from the multinomial logit. The primary difference is in the *Government Reaction* model. Using the multinomial logit model, it appears that PPC and Conservative supporters are much less likely to say the government’s response was appropriate compared to Liberals and to be more likely to say the response was an underreaction as well as more likely to say it was an overreaction. This pattern is less evident in the ordered probit results. We think the difference is because the that PPC and Conservative supporters do not view the scale as ordinal. Rather they interpret the question as reading government approval, with appropriate at the most approving and both significant overreaction and Significant underreaction as the least approving.
